# Validation of a scale to measure fathers’ confidence in supporting breastfeeding

**DOI:** 10.1590/1806-9282.20241270

**Published:** 2024-12-02

**Authors:** Mariana Frassetto Velho, Maria Antônia Vicente de Camargo, Eliane Traebert, Thaise Cristina Brancher Soncini, Gabriel Oscar Cremona-Parma, Nicole Morem Pilau Moritz, Jefferson Traebert

**Affiliations:** 1 Universidade do Sul de Santa Catarina, School of Medicine – Palhoça (SC), Brazil.; 2 Universidade do Sul de Santa Catarina, Post-Graduation Program in Health Sciences – Palhoça (SC), Brazil.; 3 Maternidade Carmela Dutra – Florianópolis (SC), Brazil.

**Keywords:** Breastfeeding, Child health, Father, Cross-cultural comparison

## Abstract

**OBJECTIVE:**

The objective of this study was to describe the process of cross-cultural adaptation of the Breastfeeding Self-Efficacy Scale—Short Form among Fathers for use in Brazil.

**METHODS:**

This is a cross-cultural adaptation study that began with the translation from the original English into Portuguese, followed by back translation. A committee of experts evaluated the semantic, idiomatic, experiential, and conceptual equivalences. The pre-test of the Brazilian version of the Breastfeeding Self-Efficacy Scale—Short Form among Fathers was conducted with 10 fathers whose children were in the breastfeeding period. To assess the psychometric properties, a cross-sectional study was carried out involving 131 fathers with children in the breastfeeding phase. Factor analysis with principal component extraction and item response theory were used in data analysis.

**RESULTS:**

The Brazilian version of the Breastfeeding Self-Efficacy Scale—Short Form among Fathers presented an overall Cronbach α of 0.852. Very closely distributed factors explained 55.6% of the total variance in the principal component analysis. The item response theory showed that all questions have discriminatory characteristics.

**CONCLUSION:**

The cross-cultural adaptation process was carried out within validated international parameters and represents a potential instrument for promoting maternal and child health in Brazil.

## INTRODUCTION

Early, exclusive, and continued initiation of breastfeeding is among the most effective interventions to reduce maternal and infant morbidity and mortality rates^1^. Despite this, exclusive breastfeeding rates have increased only slightly over the past 20 years across different regions of the world^1^. In Brazil, the prevalence of exclusive breastfeeding among children under four months old was 59.7%, and for those under six months, it was 45.8%^2^.

Breastfeeding is a complex phenomenon influenced by multiple variables, where physiological factors have been shown to influence only in the short term, while sociocultural variables affect medium- and long-term outcomes^3^. Thus, in Brazil, the factors that most frequently lead to early weaning are related to habits, beliefs, and socioeconomic aspects^4^.

Several studies have highlighted the importance of a support network, especially the active participation of the father, in promoting breastfeeding and preventing early weaning^5,6,7^. A father who shares responsibilities and is present in the breastfeeding process reassures the mother of her desire to continue breastfeeding when she feels insecure or considers stopping, reinforcing her confidence and valuing her dedication^5^. This can be encouraged through health education and guidance aimed at the male population, facilitated by health professionals and public policies^5,8^. Including the father in the guidance plan for the arrival of a child is an important support tool for child health care policies, in addition to strengthening the father–child bond^7^.

In 2018, Dennis et al.^9^ validated a scale to measure fathers’ confidence in supporting their partners with breastfeeding, naming it the Breastfeeding Self-Efficacy Scale—Short Form among Fathers (BSES-SFF), consisting of 14 questions in a single dimension. Brazil currently lacks a similar scale for this purpose, and it is believed that the cross-cultural adaptation of such an instrument will be relevant in aiding the promotion of maternal and infant health in Brazil. Therefore, the present study aimed to describe the process of cross-cultural adaptation of the BSES-SFF for use in Brazil.

## METHODS

The methodological approach consisted of two distinct phases: the cross-cultural adaptation process itself and a cross-sectional epidemiological study to assess the psychometric properties of the proposed Brazilian version [Brazilian version of the Breastfeeding Self-Efficacy Scale—Short Form among Fathers (BSES-SFF-Br)].

The cross-cultural adaptation process followed the guidelines proposed by Wild et al.^10^: (1) Preparation and planning of the research through a rigorous research project detailing all steps. (2) Translation from the original English language to Portuguese, conducted by two qualified and independent translators, one being Brazilian and the other a native English speaker. (3) Reconciliation, where the two translations were compared and synthesized into a single version. (4) Back-translation, performed by an English teacher who is a native English speaker with no medical knowledge and unfamiliar with the original scale. This step served to highlight and investigate discrepancies between the original and reconciled translations, which were then revised during the problem-resolution process. (5) Harmonization, in which the translations and back-translation were compared to each other and the original instrument. This was carried out by a panel of experts consisting of researchers, an obstetrician, and two epidemiologists. They evaluated semantic, idiomatic, experiential, and conceptual equivalences. After discrepancies were identified and discussed, a pre-final version was proposed. (6) Cognitive debriefing to test the instrument, which was conducted by administering the pre-final version to 10 men who had recently become fathers, attended postpartum consultations with a pediatrician, and whose children were being breastfed. The goal was to test alternative wording and verify the comprehensibility, interpretation, and cultural relevance of the translation. (7) Review of cognitive debriefing results with final adjustments made as necessary. (8) A written report documenting the development process was completed by the researchers, who approved the proposed Brazilian version (BSES-SFF-Br).

To assess the psychometric properties of the BSES-SFF-Br, a cross-sectional epidemiological study was conducted. The study population comprised 131 men^11^, whose children were being breastfed. Eligible men were invited to participate while attending pediatric follow-up consultations at the Pediatric Clinic of the Medical Specialties Outpatient Clinic of the Universidade do Sul de Santa Catarina in the municipality of Palhoça/SC.

A non-probabilistic sample was cumulatively selected on data collection days. After the study’s objectives were presented and consent was obtained, participants were provided with an informed consent form and a research questionnaire containing the BSES-SFF-Br questions. The forms were completed by the fathers themselves under the supervision of the researchers.

Data analysis was performed using SPSS version 18.0 (SPSS Inc., Chicago, Ill., USA). To assess the reliability of the scale, internal consistency was measured using Cronbach’s alpha. Content validity was qualitatively analyzed by a panel of experts. Construct validity was assessed through two strategies. The first was exploratory factor analysis^12^, considering the adequacy of the data set obtained from the collection, using the linear correlation matrix, the Kaiser-Meyer-Olkin (KMO) test, and Bartlett’s sphericity test^12^. The Kaiser criterion for eigenvalues greater than or close to one and the scree plot were used to define the number of extracted factors. To minimize the number of questions with high loadings on each factor, principal component extraction with varimax rotation was used to define the communalities of the questions in the proposed Brazilian version^12^. A minimum factor loading of 0.5 was used as a parameter for inclusion in the model^13^.

The second strategy of analysis was based on Item Response Theory (IRT)^14^ using the JAMOVI 2.0 software. Response probability, standard error, and test information graphs were analyzed. Additionally, item-specific graphs were examined for discrimination and difficulty characteristics.

The research project was approved by the Research Ethics Committee of the Universidade do Sul de Santa Catarina under Protocol No. 4.165.533.

## RESULTS

The BSES-SFF-Br was developed following the international protocol. Semantic, idiomatic, experiential, and conceptual equivalences were discussed and evaluated by the panel of experts, resulting in the pre-final version of the scale. This version was administered to 10 men with breastfeeding children, who provided feedback on any difficulties encountered in responding. Following this process, the researchers approved the posttest Brazilian version.

A total of 131 men completed the posttest version in the cross-sectional study to assess psychometric properties. Participants’ ages ranged from 21 to 55 years, with a mean age of 34.5 years [standard deviation (SD) 6.1].

In reliability analysis, the BSES-SFF-Br demonstrated an overall Cronbach alpha of 0.852. The correlation matrix analysis showed a linear correlation among most questions (p<0.001). The KMO measure of sampling adequacy was 0.839. Bartlett’s sphericity test also indicated suitability for conducting exploratory factor analysis (p<0.001).

**Figure 1:**
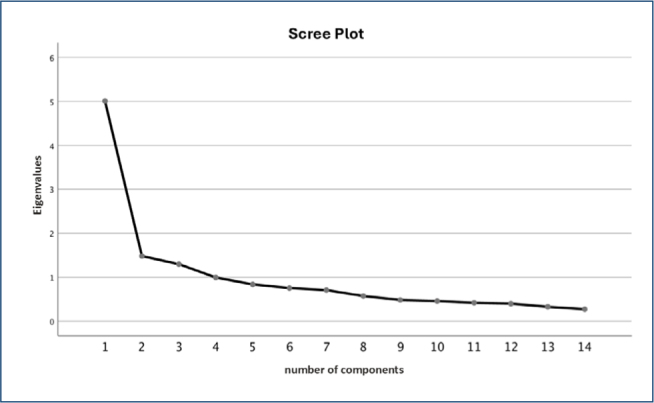
Scree plot of the latent factor values according to the order of extraction.

The analysis of communalities showed that all questions shared a significant percentage of variance with the defined factors. Based on the Kaiser criterion, only factors with eigenvalues greater than or very close to one (λ≥1) were considered. These closely distributed factors explained 55.6% of the variance. A similar pattern was observed in the scree plot (Figure 1).

The varimax rotation of factors aimed to minimize the number of variables with high loadings on one factor and maximize the variation among the weights of each principal component. Factorial loadings’ evaluation showed that all instrument items should be considered as they presented a minimum level of 0.5 (Table 1).

**Table 1:** Rotated component matrix of the Brazilian version of the Breastfeeding Self-Efficacy Scale—Short Form among Fathers (n=131).

Items	Factor loading		
	Factor 1	Factor 2	Factor 3
11. Eu consigo ajudar a mãe a terminar de amamentar o bebê em uma mama antes de mudar para a outra.	0.740		
6. Eu consigo ajudar na amamentação, mesmo que o bebê esteja chorando.	0.705		
12. Eu consigo ajudar a mãe a manter a amamentação no seio em todas as mamadas.	0.676		
4. Eu consigo ajudar a mãe a garantir que a pega do nosso bebê esteja correta durante toda a alimentação.	0.665		
5. Eu consigo ajudar a mãe a lidar com a amamentação para nossa satisfação.	0.630		
13. Eu consigo ajudar a mãe a dar conta de amamentar sempre que o bebê precisar.	0.595		
3. Eu consigo ajudar a mãe a amamentar o bebê para que não seja necessário o uso de complemento.	0.547		
7. Eu consigo estimular que a mãe queira continuar amamentando o bebê.	0.445		
9. Eu consigo ficar satisfeito com a experiência da amamentação.		0.704	
10. Eu consigo lidar com o fato de a amamentação tomar muito tempo.		0.652	
8. Eu consigo deixar a mãe confortável para amamentar mesmo que na presença de outros familiares.		0.649	
2. Eu consigo lidar bem com a amamentação. assim como em outras tarefas.		0.621	
1. Eu consigo saber quando o bebê está recebendo leite suficiente da mãe.			0.805
14. Eu consigo saber quando o bebê está satisfeito com a amamentação.			0.781

**Figure 2:**
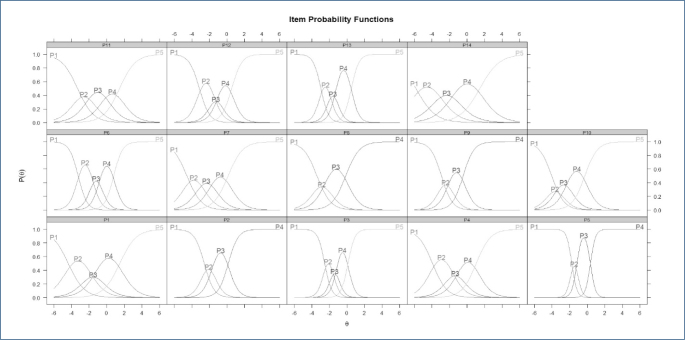
Distribution curves for the possible responses to the Brazilian version of the Breastfeeding Self-Efficacy Scale—Short Form among Fathers questions.

Regarding analysis using IRT, the lowest Akaike information criterion indicated that the data fit best with the graded response model. The skill level ranged from 20 to 70% of the total test score. Reliability was higher for average or below-average skill levels. The provided information was greatest at a skill level below average (-2) and close to 0.5. The distribution of response curves for possible question answers is shown in Figure 2. All questions exhibited discriminatory characteristics, contributing to the adequacy of the proposed Brazilian version. Questions 3, 5, and 13 provided the most information, and questions 3, 5, 6, and 13 showed the highest difficulty levels.

## DISCUSSION

In recent years, many studies have highlighted the importance of paternal involvement in maintaining breastfeeding^15,16,17^. A study conducted in Ceará^7^ on paternal knowledge and attitudes regarding the importance of breastfeeding showed that men have limited knowledge about breastfeeding benefits. Given the significance of this context, this study is the first to provide a tool for measuring Brazilian fathers’ self-efficacy in supporting their partners with breastfeeding.

The BSES-SFF-Br demonstrated an overall Cronbach alpha of 0.852, similar to the original instrument’s value of 0.91^9^, indicating good internal consistency of the adapted instrument. Similarly, studies with African^18^ and Turkish^19^ fathers using the same instrument found overall Cronbach alpha coefficients of 0.92 and 0.93, respectively. This indicates and reinforces the applicability of the BSES-SFF scale in different cultures, although studies with populations of different characteristics are needed to confirm these findings.

Regarding construct validity, the tests performed demonstrated the correlation of all questions with the theme, and the evaluation of factor loadings showed that all should be considered. This corroborates the findings and structure of the original scale^9^, as seen in the African^18^ and Turkish^19^ studies. The distributed factors explained 55.6% of the variance, slightly different from the original article’s finding (48.9%)^9^. Therefore, the BSES-SFF-Br demonstrated psychometric properties of validity and reliability for this population, capable of measuring paternal self-efficacy in promoting breastfeeding.

These findings can contribute to improving public policies for child health, especially those targeting breastfeeding, and emphasize the importance of the support network and family participation^20^ in reducing early weaning.

However, this study has some limitations. The generalization of results should be considered with caution due to the small sample size. Additionally, the entire sample came from a single region of the country. Therefore, we suggest conducting studies with different cultural and demographic characteristics in Brazil to understand the relevance of the instrument in other regions.

## CONCLUSION

The cross-cultural adaptation process followed all international guidelines and recommendations, resulting in a translated, adapted, and culturally appropriate instrument for use in Brazil.

Based on the performed analyses, the BSES-SFF-Br demonstrated good validity and reliability, enabling its use in similar population profiles.

Thus, the instrument may contribute to health promotion by identifying and developing actions and strategies aimed at paternal involvement in supporting breastfeeding. However, new studies with larger and more diverse samples are necessary to confirm the instrument’s applicability in different contexts.

## Data Availability

The database that originated the article is available upon request, with the corresponding author.
